# Development and internal validation of a preliminary nomogram for the early prediction of chronic ankle instability following a first-episode lateral ankle sprain with acute-phase MRI indication

**DOI:** 10.3389/fspor.2026.1803053

**Published:** 2026-05-25

**Authors:** Donglong Shang, Yuankai Zhang, Yan Zhao, Lihong Fan

**Affiliations:** 1 Department of Orthopaedics, Xi'an No.1 Hospital, Northwest University, Xi’an, Shaanxi, China; 2 Department of Orthopaedics, The Second Affiliated Hospital of Xi’an Jiaotong University, Xi’an, Shaanxi, China

**Keywords:** chronic ankle instability, lateral ankle sprain, MRI, prediction nomogram, risk factors

## Abstract

**Introduction:**

Chronic ankle instability (CAI) is a common sequela following acute lateral ankle sprain (LAS). In order to treat acute lateral ankle sprains more effectively, it is important to recognize patients who are at high risk of developing CAI. The aim of this study is to develop a Nomogram to predict the risk of developing CAI after the onset of the first LAS.

**Method:**

This study included 181 patients diagnosed with their first LAS between December 1, 2017, and December 1, 2020, who received plain radiograph and MRI scanning within the first 2 weeks after LAS. Independent risk factors for CAI post-initial LAS were determined through separate univariate and multivariate analyses. The clinical utility and predictive efficacy of the nomogram were evaluated using the C-index, area under the receiver operating characteristic (ROC) curve, calibration curves, and decision curve analysis. Internal validation was assessed by bootstrap validation.

**Results:**

Multivariate analysis identified five significant predictors for CAI: age ≤ 23 years, body mass index (BMI) of 24–28 kg/m² and BMI ≥28 kg/m², injury to the posterior talofibular ligament, a large bone marrow lesion of the talus, and Grade 2 tibial talar joint effusion. The prediction model demonstrated good discrimination, with an apparent C-index of 0.831 and an AUC of 0.818. Bias-corrected internal validation yielded a C-index of 0.816. The calibration curve showed strong agreement between predicted probabilities and actual outcomes.

**Conclusion:**

Pending external validation in diverse prospective cohorts, this internally validated preliminary nomogram demonstrates potential to predict the risk of CAI development after a first episode of LAS in patients who underwent acute-phase MRI scanning. This tool may help identify high-risk patients but requires rigorous external validation before clinical implementation.

## 1 Introduction

Lateral ankle sprains (LAS) are among the most common musculoskeletal injuries in young and physically active populations (1), affecting over 40% of the general population at some point during their lifetime (2, 3). The high prevalence of LAS injuries imposes significant socio-economic burdens, including substantial costs associated with initial management and rehabilitation, and reductions in labor productivity (4, 5). Despite interventions such as taping and physical rehabilitation, 30%–40% of LAS patients may develop chronic ankle instability (CAI) (6), which implies that some important prognostic factors associated with the efficacy of rehabilitation remain unknown. Sequelae associated with CAI, such as pain, persistent swelling, the ankle “giving-way”, recurrent injury risk, and impaired joint function, significantly contribute to the overall social and economic costs (6, 7). Consequently, the assessment and mitigation of CAI risk post-acute LAS represent critical priorities (8). Moreover, patients experiencing severe CAI typically necessitate surgical interventions to prevent recurrent ankle sprains and subsequent long-term osteoarthritis (9). Early surgical interventions are often more effective in achieving superior functional outcomes compared to later repairs, particularly following simultaneous ligament ruptures and laxity post-initial LAS episode (10). At present, no established tool exists to immediately assess the likelihood of CAI development following an acute LAS episode (11). Identifying patients who would benefit from early surgical intervention is also crucial.

Early risk stratification at the time of initial diagnosis enables clinicians to identify high-risk patients who may benefit from closer monitoring, targeted counseling, and more intensive follow-up. Such an approach allows for timely adjustments to treatment strategies, potentially slowing disease progression and improving patient quality of life.

A variety of factors have been studied, including demographic characteristics, severity of LAS, foot and ankle anatomy, neuromuscular factors, and initial treatment, which may play an important role in the development of CAI (12–14). Although these clinical features provide some insight into the assessment of the risk of progression of CAI after LAS, there is currently no comprehensive risk model to quantitatively assess the likelihood of progression in patients with the first LAS. Developing predictive models that integrate multiple risk factors is essential for accurately estimating the likelihood of progression from LAS to CAI. Such models could facilitate early identification of high-risk individuals, enabling timely and tailored interventions.

The purpose of this study was to identify and assess the risk factors associated with the development of CAI after a first LAS, to construct predictive models and to evaluate their accuracy and validity. In addition, clinical decision curve analysis was used to assess the clinical value of the developed models.

## 2 Method

### 2.1 Research design

This investigation was conducted as a retrospective cohort study of patients who presented to the emergency department and reported the first ankle inversion injury in their lifetime and underwent radiographs of the injured ankle on flat films and MRI scans within two weeks of the injury. The standard-of-care protocol for LAS patients during the study period involved initial rest, ice, compression, and elevation (RICE), with early mobilization as tolerated. Referral to a physiotherapy program was made at the discretion of the attending physician based on symptom severity and functional limitations. However, detailed documentation of patient adherence to these recommendations was not available for analysis. The onset of CAI was assessed by data collected through the Cumberland Ankle Instability Tool (CAIT) during the final follow-up. Demographic variables and other relevant clinical variables, including age, gender, body mass index (BMI), and treatments, were recorded.

### 2.2 Participants

From December 1, 2017, to December 1, 2020, all patients who presented to the emergency department or outpatient clinic with a first occurrence of LAS and underwent an MRI scan during the acute phase of the injury (defined as within 2 weeks of the first injury) were considered eligible. Of note, because of patient anxiety regarding injury severity and the relatively low out-of-pocket cost of MRI scans in our local healthcare setting (approximately 500 yuan), clinicians at our institution frequently ordered acute-phase MRI scans for LAS patients. This practice was more liberal than that recommended by standard clinical decision rules, such as the Ottawa Ankle Rules. This practice introduces a selection bias, as our cohort is likely skewed toward more severe presentations. Patient exclusion criteria included: (1) previous history of two or more ankle sprains; (2) failure to complete at least 2 years of follow-up; (3) absence of frontal and lateral radiographs of the ankle joint; (4) fracture, including avulsion fracture; (5) any scarring of the ligaments of the ankle joint that have become thinned or thickened, but not have discontinuous or periligamentous edema; (6) presence of any bony or soft-tissue tumor in the foot or ankle; (7) previous surgery on the ankle joint or open trauma; (8) refusal to participate in the clinical study. The entire patient cohort in this study was of Chinese descent.

### 2.3 Ethics statement

The study was conducted in accordance with the Declaration of Helsinki, and approved by the Ethics Committee of Xi'an Jiaotong University.

### 2.4 Baseline characteristics

Demographic data, including age, gender, and body mass index (BMI), were systematically recorded. Age was dichotomized at the median of the study cohort (23 years). BMI was categorized according to the Chinese BMI classification standard into three groups: <24 kg/m^2^ (normal or underweight), 24–28 kg/m^2^ (overweight), and ≥28 kg/m^2^ (obese). To evaluate the severity of ligamentous injuries, a delayed physical examination was conducted 4–7 days following the initial LAS, including an anterior drawer test [to check the function of the anterior talofibular ligament (ATFL)] and a talocalcaneal inversion tilt test [to check the function of the anterior talofibular ligament and the calcaneofibular ligament (CFL)]. Ankle swelling was assessed using figure-of-eight measurements (15). For final follow-up patient-reported outcomes, the CAIT, a well-validated self-report tool for assessing CAI, was utilized. The CAIT is a 9-item, 30-point scale used to diagnose and grade ankle stability (16).

### 2.5 Radiological evaluation

#### 2.5.1 MRI protocol

All MRI examinations were performed on a 3.0 T superconducting MR scanner (MAGNETOM Skyra, Siemens Healthcare, Erlangen, Germany) with a dedicated 16-channel ankle coil. The routine ankle MR protocol included the following sequences: (1) Axial fast spin-echo (FSE) proton density-weighted (PDw) sequence (TR/TE = 3500/33 ms, slice thickness = 3.0 mm, interslice gap = 0.3 mm, FOV = 14 cm, matrix = 512 × 256, ETL = 12); (2) Coronal FSE PDw sequence (TR/TE = 3500/33 ms, slice thickness = 3.5 mm, interslice gap = 0.3 mm, FOV = 14 cm, matrix = 512 × 384, ETL = 12); (3) Sagittal FSE PDw sequence (TR/TE = 3000/33 ms, slice thickness = 3.0 mm, interslice gap = 0.3 mm, FOV = 14 cm, matrix = 512 × 384, ETL = 12); (4) Sagittal FSE short tau inversion recovery (STIR) sequence (TR/TE = 4000/13 ms, TI = 170 ms, slice thickness = 3.0 mm, interslice gap = 0.3 mm, FOV = 18 cm, matrix = 256 × 192, ETL = 12). Axial and coronal fat-suppressed T2-weighted sequences were also acquired when clinically indicated. All images were anonymized and independently reviewed by three musculoskeletal radiologists (each with >8 years of experience) who were blinded to all clinical data and final outcomes.

#### 2.5.2 Image evaluation and consensus process

Ligaments evaluated in this study included: the anterior talofibular ligament (ATFL), calcaneofibular ligament (CFL), posterior talofibular ligament (PTFL), medial collateral ligament (deltoid ligament), inferior tibiofibular coalition ligament, interosseous membrane, spring ligament complex, and tarsal sinus ligament injuries. ATFL and CFL injuries are common and can be classified as Normal (grade 0), Low-grade sprains (grade 1): periligamentous edema only with no disruption of fibers, Partial disruptions (grade 2): partial disruption with preservation of residual fibers, Complete disruptions (grade 3): complete disruption (17). Damage to other ligamentous structures is Relatively uncommon and therefore graded as Grade 0 = normal, Grade 1 = present. Acute bone marrow lesions (BMLs) are defined as edema adjacent to the subchondral bone plate with or without cartilage surface damage. The grading of BMLs in the tibia, fibula, calcaneus, and navicular bones is simplified: Grade 0 = normal, Grade 1 = present. BMLs of the talus were assessed primarily as ordinal variables according to previously published classifications (18). Grade 0 = normal, Grade 1 = small subchondral edema only, Grade 2 = large subchondral edema only. Small subchondral edema of the talus is defined as being confined to only one part of the talus (body, neck, or head), while large talar subchondral edema is defined as involving 2 or 3 areas of the talus. A simple bone contusion is defined as edema that does not involve the subchondral region. Effusions in the tibiotalar and talocalcaneal joints were graded: Grade 0 = physiologic effusion (normal); Grade 1 = <50% of maximum capsular distension; Grade 2 = ≥50% of maximum capsular distension (19). After independent evaluation, all discrepancies were resolved through a consensus reading session where the three evaluators jointly reviewed the images and reached a unanimous final grading for each variable. The consensus results were used for all subsequent analyses.

#### 2.5.3 Inter-rater reliability

Inter-rater reliability for the primary MRI findings was assessed using the intraclass correlation coefficient (ICC) with a two-way mixed-effects model for absolute agreement. The ICC values indicated excellent reliability for talar bone marrow lesion grade (ICC = 0.89; 95% CI: 0.83–0.93), PTFL injury (ICC = 0.92; 95% CI: 0.87–0.95), and tibiotalar joint effusion grade (ICC = 0.85; 95% CI: 0.78–0.90).

### 2.6 Definitions of CAI and patient-reported outcomes

Given the ongoing debate over the definitive diagnosis of CAI, this study employed a widely accepted definition. Specifically, a diagnosis of CAI was established if a patient met any one of the following three criteria at the final 24-month follow-up: (a) CAIT < 24; (b) CAIT ≥ 24 with ankle weakness;(c) CAIT < 26 with a history of recurrent ankle sprains, with at least one persistent symptom (pain, swelling, and weakness) at the 24-month follow-up visit (20, 21). The final outcome of CAI diagnosis was determined by a majority consensus of 3 independent evaluators.

### 2.7 Development and evaluation of nomogram

Univariate analysis was employed to examine the relationship between clinical characteristics and progression of CAI after LAS. Multivariate logistic regression was employed to assess independent predictors of CAI progression (entry criterion: *P* < 0.050). Subsequently, a nomogram predicting the probability of risk of CAI after LAS was created. The nomogram visually represents predicted probabilities on a scale from 0 to 100, facilitating clinical use.

The total points assigned to each predictor reflect its relative contribution to the predicted probability. The predictor with the largest absolute beta coefficient was assigned 100 points, and the remaining predictors received proportionally fewer points.

To estimate an individual patient's risk of developing CAI, the points for each predictor are summed, and a vertical line is drawn from the total points axis to the risk axis. The ability of the nomogram to discriminate the risk of CAI after LAS was evaluated using the Receiver operating characteristic (ROC) curve, the area under the ROC curve (AUC), and the consistency index (C-index). A nonparametric bootstrap of 1000 resamples was performed and calibration curves were created to assess the consistency between actual observations and nomogram predictions. Decision curve analysis (DCA) assesses the clinical utility of nomogram by quantifying the net benefit at different threshold probabilities. DCA evaluates the clinical utility of a predictive model by combining measures of accuracy (e.g., sensitivity and specificity) with decision-analytic principles. DCA constructs the curves by taking into account both the sensitivity and specificity of the data set. By assessing net clinical benefit, DCA allows different strategies for patient intervention to be compared with the default strategy of intervening in all or no patients (22).

### 2.8 Statistical analysis

Statistical analyses were conducted using SPSS 23.0, R version 4.3.1, and the Rstudio data package. Continuous variables are expressed as mean ± standard deviation and ordinal/categorical variables are expressed as numbers (%). For each categorical/sequential variable, statistical significance was assessed using the Pearson chi-square test or Fisher's exact test (if the expected count for either contingent unit was less than 5). For each continuous variable, statistical significance was tested using the Mann–Whitney *U*-test. When the area under the curve was greater than 0.5, receiver operating characteristic analysis was used to determine the threshold value for converting continuous variables to categorical variables. When the univariate analysis yielded a *p* value of less than 0.2, the variable was further included in logistic regression for multivariate analysis to obtain independent risk factors for the development of CAI after LAS, and a *P* < 0.05 was considered a statistically significant difference (23). With 65 events and 5 predictors included in the final multivariate model, the events-per-variable (EPV) was 13, which is above the commonly recommended threshold of 10, indicating adequate stability of the regression model. Age was dichotomized at the median for simplicity in the nomogram after initial analysis revealed a non-linear relationship with CAI risk. To further evaluate the appropriateness of this approach, we conducted an exploratory analysis modeling age as a continuous variable using restricted cubic splines (RCS) with 3 knots (located at the 10th, 50th, and 90th percentiles) (24). The Wald test was used to assess the significance of the non-linear component. Model fit was compared using the Akaike Information Criterion (AIC). The dichotomized model was retained as the primary model for nomogram construction based on three considerations: (1) superior clinical interpretability for rapid bedside risk assessment in acute care settings, where a clear binary threshold defining “young” patients at elevated risk facilitates immediate clinical decision-making; (2) compatibility with the visual nomogram format, where a simple binar*y* axis is more readily actionable than a complex non-linear scale; and (3) consistency with existing literature on CAI risk prediction, which frequently reports age thresholds for clinical decision-making (25). We acknowledge that dichotomization leads to a loss of information and that future studies should explore more sophisticated methods, such as spline functions, to model continuous predictors. This manuscript was prepared in accordance with the Transparent Reporting of a multivariable prediction model for Individual Prognosis Or Diagnosis (TRIPOD) statement.

## 3 Results

### 3.1 General characteristics

We collected data from a total of 202 patients, 21 of whom were excluded due to ineligibility or incomplete data. Among the 21 excluded patients, 5 had a history of two or more previous ankle sprains, 3 had no complete ankle imaging, 4 had fractures including avulsion fractures, 2 had previous ankle surgeries, 4 refused to participate, 3 did not complete at least a 2-year follow up. This study's final analysis included a cohort of 181 patients. There were no missing data for any of the variables included in the final analysis for these 181 patients, and thus no data imputation methods were required. The average age of participants was 24.5 ± 6.5 years, with females comprising 40.3% of the cohort (*n* = 73). The average interval between injury and MRI scanning was 3.1 ± 3.9 days. The mean duration of follow-up was 3.0 ± 0.6 years. Of the cohort, 65 patients (36.2%) developed chronic ankle instability (CAI) following their first lateral ankle sprain (LAS). Table 1 presents a summary of the univariate analysis of demographic and clinical characteristics. Based on predefined statistical criteria, variables including age, body mass index, CFL injury, PTFL injury, tibial talar joint effusion, and bone marrow lesion of the talus (within 2 weeks), which all had *P*-values less than 0.2, were included in the multivariate analysis (Table 1). Multivariate Logistic analysis (Table 2) revealed associations between CAI events and specific risk factors: age ≤ 23 years [odds ratio (OR) = 2.13, 95% confidence interval (95% CI): 1.21–3.74, *P* = 0.008]; body mass index 24–28 kg/m^2^ (OR = 1.78, 95% CI: 1.11–2.86, *P* = 0.017); and body mass index ≥ 28 kg/m^2^ (OR = 2.09, 95% CI: 1.20–3.63, *P* = 0.009); posterior talofibular ligament injury (OR = 2.17, 95% CI: 1.05–4.48, *P* = 0.035); large bone marrow lesion of the talus (within 2 weeks) (OR = 2.69, 95% CI: 1.30–5.58, *P* = 0.008), as well as a Grade 2 tibial talar joint effusion (OR = 2.61, 95% CI: 1.39–4.89, *P* = 0.003).

**Table 1:** Univariate analysis of demographic and clinical characteristics between the LAS and CAI groups.

Variable	LAS (*n* = 116)	CAI (*n* = 65)	*P*
Age(years)			0.010
>23	65 (56.0)	21 (32.3)	
≤1	51 (44.0)	44 (67.7)	
BMI (kg/m²)			0.011
≥11	34 (29.3)	20 (30.8)	
24–28	67 (57.8)	32 (49.2)	
<24	15 (12.9)	13 (20.0)	
Follow-up duration (years)	3.0 ± 0.6	3.0 ± 0.6	0.352
Gender			0.795
Male	70 (60.2)	38 (58.8)	
Female	46 (39.8)	27 (41.2)	
10-meter walk test			0.657
<10 s	66 (57.1)	39 (59.5)	
≥10 s	50 (42.9)	26 (40.5)	
Grades of anterior drawer test			0.889
0	70 (60.6)	38 (58.8)	
1	36 (31.2)	22 (33.6)	
2 or 3	10 (8.2)	5 (7.6)	
Grades of inversion tilt test			0.583
0	96 (82.7)	51 (78.6)	
1	18 (15.6)	13 (19.8)	
2 or 3	2 (1.7)	1 (1.6)	
Swelling measured by figure-of-eight (mm)	13.3 ± 8.1	14.0 ± 8.5	0.425
Posterior talar glide test (°)	1.66 ± 1.15	1.53 ± 1.00	0.269
Grades of ATFL injury			0.991
0 ((normal)	12 (10.4)	6 (9.9)	
1 (only edema)	43 (36.8)	25 (38.2)	
2 (partial tear)	39 (33.3)	21 (32.1)	
3 (complete tear)	22 (19.5)	13 (19.8)	
Grades of CFL injury			0.190
0 (normal)	70 (60.2)	41 (62.6)	
1 (only edema)	22 (19.0)	9 (14.5)	
2 (partial tear)	22 (19.0)	11 (17.6)	
3 (complete tear)	2 (1.8)	4 (5.3)	
Presence of PTFL injury			0.054
Yes	10 (8.7)	10 (15.3)	
No	106 (91.3)	55 (84.7)	
Presence of medial collateral ligament injury			0.584
Yes	10 (8.2)	6 (9.9)	
No	106 (91.8)	59 (90.1)	
Presence of syndesmotic ligament injury			0.225
Yes	18 (15.6)	13 (20.6)	
No	98 (84.4)	52 (79.4)	
Presence of interosseous membrane injury			0.481
Yes	3 (3.0)	1 (2.3)	
No	113 (97.0)	64 (97.7)	
Presence of spring ligament complex injury			0.383
Yes	5 (4.3)	2 (3.1)	
No	111 (95.7)	63 (96.9)	
Presence of sinus tarsi ligament injury			0.446
Yes	3 (3.0)	3 (4.6)	
No	113 (97.0)	62 (95.4)	
Bone marrow lesion of talus			<0.001
0 (normal)	37 (32.0)	12 (18.3)	
1 (involving 1 part of the talus)	64 (55.0)	32 (48.9)	
2 (involving 2 or 3 parts of the talus)	15 (13.0)	21 (32.8)	
Bone marrow lesion of calcaneus			0.249
Yes	10 (8.7)	3 (5.3)	
No	106 (91.3)	62 (94.7)	
Bone marrow lesion of fibular			0.452
Yes	6 (5.6)	5 (7.6)	
No	110 (94.4)	60 (92.4)	
Bone marrow lesion of tibial			0.607
Yes	14 (11.7)	6 (9.9)	
No	102 (88.3)	59 (90.1)	
Bone marrow lesion of navicular bone			0.32
Yes	12 (10.0)	4 (6.9)	
No	104 (90.0)	61 (93.1)	
Effusion of tibiotalar joint			<0.001
0 (normal)	46 (39.8)	17 (26.0)	
1 (<50% of maximum capsular distension)	47 (40.3)	21 (32.8)	
2 (≥50% of maximum capsular distension)	23 (19.9)	27 (41.2)	
Effusion of talocalcaneal joint			0.783
0 (normal)	47 (40.7)	24 (37.4)	
1 (<50% of maximum capsular distension)	40 (34.6)	23 (35.1)	
2 (≥50% of maximum capsular distension)	29 (24.7)	18 (27.5)	

ATFL, anterior talofibular ligament; BMI, body mass index; CAI, chronic ankle instability; CFL, calcaneofibular ligament; LAS, lateral ankle sprain; PTFL, posterior talofibular ligament.

**Table 2:** Multivariable logistic regression analysis.

Variable	β Coefficient	SE	Adjusted OR	95% CI	*P*
Age (years)					
>23	–	–	1.00 (Ref)	–	–
≤23	0.756	0.287	2.13	1.21–3.74	0.008
BMI (kg/m²)					
<24	–	–	1.00 (Ref)	–	–
24–28	0.557	0.242	1.78	1.11–2.86	0.017
≥28	0.737	0.281	2.09	1.20–3.63	0.009
Grades of CFL injury					
0 (normal)	–	–	1.00 (Ref)	–	–
1 (only edema)	0.113	0.287	1.12	0.64–1.96	0.694
2 (partial tear)	0.157	0.312	1.17	0.63–2.15	0.614
3 (complete tear)	0.239	0.384	1.27	0.60–2.69	0.533
PTFL Injury					
No	–	–	1.00 (Ref)	–	–
Yes	0.775	0.371	2.17	1.05–4.48	0.035
bone marrow lesion of talus					
0 (normal)	–	–	1.00 (Ref)	–	–
1 (involving 1 part of the talus)	0.104	0.278	1.11	0.64–1.91	0.709
2 (involving 2 or 3 parts of the talus)	0.989	0.372	2.69	1.30–5.58	0.008
Effusion of tibiotalar joint					
0 (normal)	–	–	1.00 (Ref)	–	–
1 (<50% of maximum capsular distension)	0.157	0.275	1.17	0.68–2.01	0.569
2 (50% of maximum capsular distension)	0.959	0.320	2.61	1.39–4.89	0.003

BMI, body mass index; CFL, calcaneofibular ligament; PTFL, posterior talofibular ligament; OR, odds ratio; CI, confidence interval; SE, standard error; Ref, reference category. Model intercept: −2.834.

### 3.2 Nomogram development

To construct a predictive model, scores were assigned to each of the five factors based on their respective impacts on the progression from LAS to CAI. The full regression equation, including the intercept and beta coefficients for each predictor, is presented in Table 3.

**Table 3:** Full regression equation for the prediction nomogram.

Predictor	β coefficient	SE	Wald statistic	*P*-value
Intercept	−2.834	0.412	47.31	<0.001
Age rcepttr t	0.756	0.287	6.94	0.008
BMI 24–28 kg/m²	0.577	0.242	5.69	0.017
BMI ²4– kg/m²	0.737	0.281	6.88	0.009
PTFL injury (Yes)	0.775	0.371	4.36	0.035
Talar BML Grade 2	0.989	0.372	7.07	0.008
Tibiotalar effusion Grade 2	0.959	0.320	8.98	0.003

BMI, body mass index; PTFL, posterior talofibular ligament; BML, bone marrow lesion; SE, standard error.

**Figure 1:**
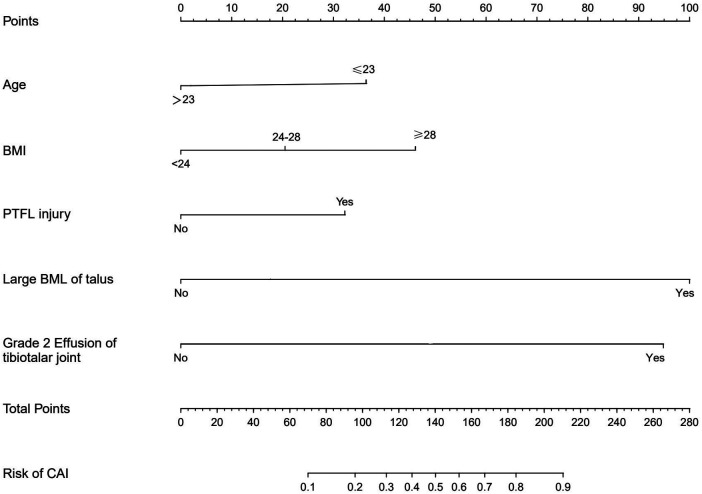
Risk nomogram for predicting CAI. This figure illustrates the probability that patients with LAS will develop CAI, based on the depicted features. To use this nomogram, follow these steps: (1) Locate the patient's value on each variable axis (e.g., for a 20-year-old patient, locate “≤23” on the Age axis); (2) Draw a vertical line upward from that point to the “Points” scale at the top of the figure to determine the score for that variable (e.g., Age ≤23 = approximately 42 points); (3) Repeat this process for all five predictors and sum all individual scores; (4) Locate the total score on the “Total Points” axis at the bottom of the figure; (5) Draw a vertical line downward to the “CAI Risk” axis to estimate the predicted probability. For example, a 20-year-old patient (Age ≤23: ∼42 points) with BMI of 26 kg/m^2^ (BMI 24–28: ∼30 points), PTFL injury (Yes: ∼38 points), Grade 2 talar BML (∼52 points), and Grade 2 effusion (∼48 points) would have a total score of approximately 210 points, corresponding to a predicted CAI risk of approximately 65%.

Employing the “rms” package within R software, a visual nomogram was constructed to depict the predictive model (Figure 1).

### 3.3 Nomogram validation

**Figure 2:**
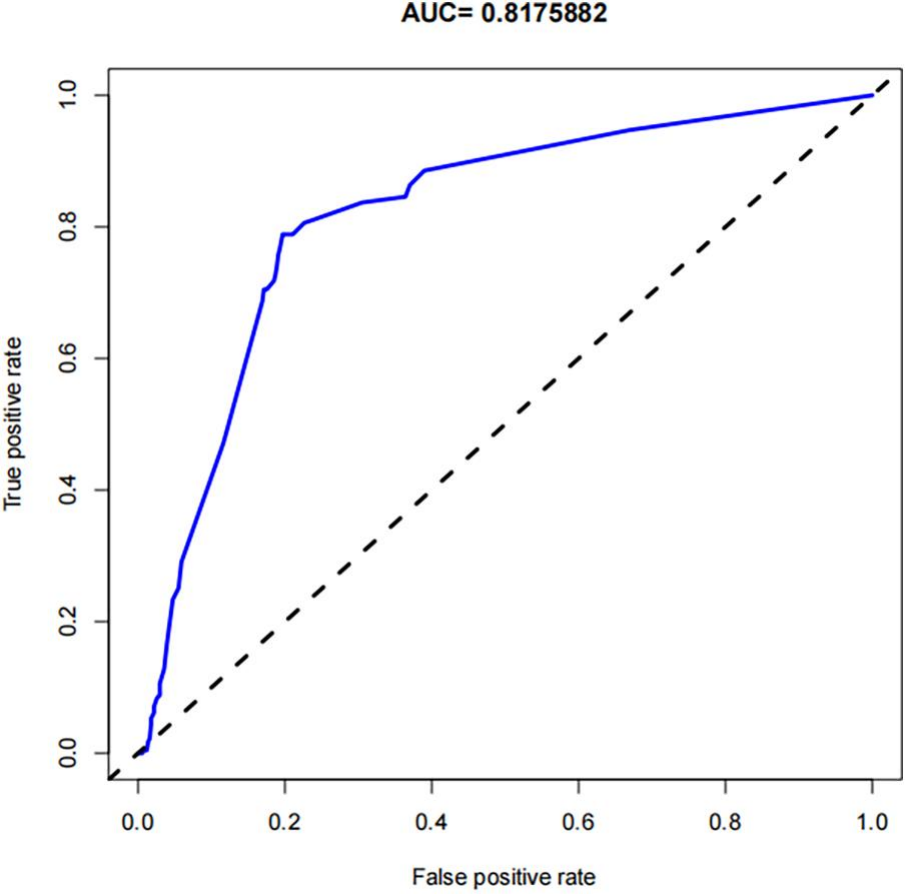
ROC curve. This figure depicts the ROC curve and AUC value for the nomogram, illustrating the model's discriminative ability. ROC, stands for Receiver Operating Characteristic; AUC, represents the Area Under the ROC Curve.

**Figure 3:**
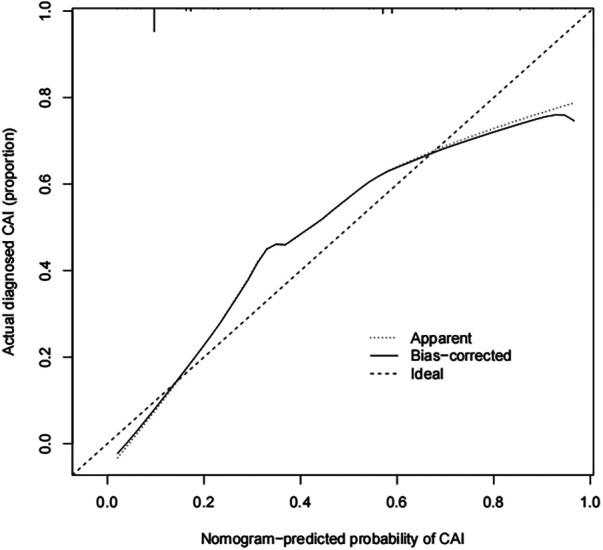
Calibration curve. This figure presents the calibration curve for the nomogram. The *x*-axis denotes the predicted risk of CAI, and the *y*-axis signifies the proportion of diagnosed CAI. The diagonal dashed line symbolizes perfect prediction by an ideal model, whereas the solid line indicates the actual performance of the nomogram. Closer alignment with the diagonal dashed line denotes better predictive accuracy.

**Figure 4:**
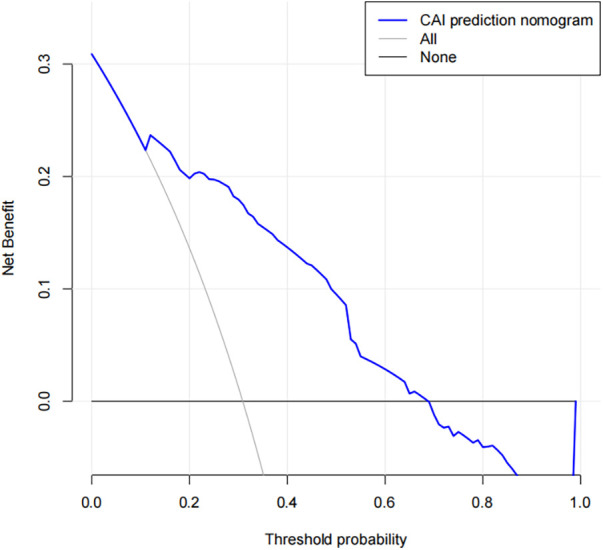
DCA curve. This figure displays the DCA curve for the nomogram, detailing the clinical net benefits at various risk thresholds. The *y*-axis quantifies the net benefit, and the blue line illustrates the DCA for predicting CAI risk with the nomogram. The thin solid line depicts a hypothetical scenario in which all patients develop CAI, while the thin bold solid line depicts a scenario where no patients develop CAI. The threshold range for DCA is derived from the training set, grounded on the model's sensitivity and specificity. Interventions target patients whose risk falls within the threshold range (>12%, <68%), where the net benefit surpasses that of either universal intervention or no intervention at all. DCA, stands for decision curve analysis; CAI, refers to chronic ankle instability.

The predictive accuracy of the model was evaluated using the area under the receiver operating characteristic curve (AUC) and the concordance index (C-index). The model demonstrated an AUC of 0.818 (95% CI: 0.785–0.851) and an apparent C-index of 0.831 (95% CI: 0.798–0.864). Internal validation using 1000 bootstrap resamples yielded a bias-corrected C-index of 0.816, indicating good discriminative ability after accounting for optimism (Figure 2). The calibration curve shows that the bias-corrected trajectory of the model closely aligns with the ideal line (Figure 3). Decision curve analysis (DCA) delineates the clinical net benefits at various risk thresholds (Figure 4), revealing that the net benefits are optimal when the threshold probability lies between 12% and 68%, surpassing the outcomes of universal or no intervention. These findings suggest that the model may offer clinical benefit when used to predict the risk of CAI following a first-episode LAS.

### 3.4 Exploratory analysis of continuous age modeling using restricted cubic splines

To assess whether modeling age as a continuous variable would improve predictive performance, we re-specified the multivariable logistic regression model using restricted cubic splines (RCS) with 3 knots. The non-linear component of the age-RCS term achieved statistical significance (Wald test for non-linearity, *P* = 0.031), confirming that the relationship between age and CAI risk deviates from linearity. The RCS-based model demonstrated an apparent C-index of 0.835, a marginal improvement over the dichotomized model (C-index = 0.831). The odds ratios for BMI, PTFL injury, talar BML, and tibiotalar effusion remained essentially unchanged. Model fit comparison using the Akaike Information Criterion (AIC) favored the RCS model over the dichotomized model, indicating modestly better fit.

Despite the statistical advantages of the RCS approach, the dichotomized model was retained as the primary model for nomogram construction because a binary age classification (≤23 vs. >23 years) is easier to interpret at the bedside, is more compatible with the standard visual nomogram format, and is consistent with reporting practices in the existing CAI literature. The RCS results are presented here for transparency and to guide future prospective studies with larger sample sizes, where continuous modeling of age should be adopted as the preferred analytic approach.

### 3.5 Sensitivity analysis of CAI definition

To evaluate the robustness of our findings to the composite CAI definition, we performed sensitivity analyses using each of the three diagnostic criteria individually. The model's discriminative performance remained stable across all three definitions: C-index for criterion (a) CAIT <24 = 0.823, (b) CAIT ≥24 with weakness = 0.812, and (c) CAIT <26 with recurrent sprain = 0.828. These consistent results support the stability of the primary analysis.

## 4 Discussion

The long-term outcomes of chronic ankle instability (CAI), such as chronic osteochondral injuries and osteoarthritis, can result in significant health complications for patients. Despite the application of standard care, including bracing and physical rehabilitation, 30%–40% of patients with lateral ankle sprains (LAS) progress to chronic ankle instability (CAI) (11). This observation suggests that additional, as yet unidentified, factors influence the pathogenesis of CAI following LAS. This study demonstrated that the presence of CAI after the first LAS was associated with the following 5 prognostic factors: age (≤23 years), body mass index (≥24 kg/m^2^), PTFL injury, massive talar bone marrow edema, and grade 2 tibio-talar joint effusion. Body mass index (BMI) and age have previously been shown to be associated with CAI outcomes after LAS (26). The present study further validated these observations and found that young age (≤23 years) and increased body mass index (BMI) (≥24 kg/m^2^) were associated with the development of CAI.

To date, numerous studies have identified a correlation between deficits in ankle proprioception and the development of CAI (27). It is widely accepted that damage to proprioceptive nerve fibers within sprained ligaments contributes to recurrent instability and the development of CAI (28). In support of this notion, previous studies have shown that ankle rehabilitation strategies targeting proprioception are highly effective in treating CAI (29). Currently, it remains unclear which patients, after LAS treatment, will have restoration of proprioceptive nerves within sprained ligaments. Previous reports have suggested that the severity of ATFL/CFL injuries is not related to the development of CAI post-LAS (30), indicating that the initial severity of proprioceptive nerve injury within sprained ligaments may not be a determining factor in the loss of ankle proprioception in CAI patients. Our study further validates this point, showing that the severity of ATFL/CFL injuries is not directly related to the development of CAI post-LAS. Interestingly, we found a closer association between PTFL injury and CAI sequelae. The PTFL is generally considered to play a secondary role in maintaining ankle stability. Biomechanical and clinical studies have demonstrated that anatomic repair of the ATFL alone is often sufficient to restore talar stability, whereas isolated PTFL injury rarely results in functional instability. In this context, PTFL injury likely serves as a marker of more extensive ligamentous damage rather than a direct mechanical contributor to CAI, making it a useful prognostic indicator (31). Further investigation is required to confirm this finding and determine its underlying mechanisms.

Notably, we observed a significant association between the occurrence of CAI post-LAS and large block BMLs in the talus and grade 2 effusion in the tibiotalar joint. BMLs are common manifestations of bone inflammation observed in various conditions, including osteoarthritis, malignant bone tumors, and bone cartilage disorders (32–34). Similarly, joint effusion is another important manifestation of synovitis and joint inflammation (35). Many preclinical and clinical studies have suggested that the inflammatory environment delays the repair of peripheral nerves following acute injuries (36). It is therefore plausible that severe post-traumatic inflammation within the ankle joint impairs the repair of proprioceptive nerve fibers, thereby contributing to the development of CAI.

Clinical examinations, including the anterior drawer test, talar tilt test, varus stress test, and posterior drawer test, possess limited predictive utility for CAI during the acute phase of LAS (37). This study reaffirms that clinical examinations are insufficient for predicting CAI outcomes post-acute LAS.

The findings of this study must be interpreted in the context of several important limitations.

First and foremost, this model has not been externally validated. The performance metrics (C-index, AUC) reported here are based on internal validation (bootstrapping) of a single, retrospectively collected cohort. Such analyses are known to potentially overestimate model performance due to overfitting. Accordingly, this study should be regarded as a derivation study that presents a preliminary prediction tool. Rigorous external validation in a geographically, ethnically, and temporally distinct prospective cohort is an absolute prerequisite before any clinical implementation can be contemplated. Second, Inevitable selection bias must be acknowledged. According to the “Ottawa Ankle Rules” (38), standard care recommends plain radiographs for only a small subset of LAS patients. However, physicians in our hospital rarely adhere to the “Ottawa Ankle Rules” and require plain radiographs for most acute LAS patients, primarily due to the lower cost of plain radiographs. Thus, we only excluded 35 cases due to the unavailability of ankle anteroposterior and lateral radiographs. Many patients tend to undergo MRI scans in the acute phase following LAS because it costs only around 500 yuan, which is still affordable for Chinese patients (39). There is a significant risk of selection bias stemming from our inclusion criteria. By mandating an MRI scan within two weeks of injury, our cohort is likely enriched with patients who had more severe pain, swelling, or functional impairment. Patients with mild LAS who recovered quickly were less likely to seek or be offered an MRI. Consequently, our model's predictions are likely most valid for a population of LAS patients with injuries severe enough to warrant advanced imaging, and may not be generalizable to all first-time LAS presentations. The clinical context of low-cost MRI availability in our setting, while a pragmatic reality for this study, further limits its direct applicability in healthcare systems with more restricted imaging access.

Third, the generalizability of the model is constrained by the homogeneity of our cohort. The study population consisted solely of Chinese Han patients from a single orthopedic center. The predictive value of specific risk factors, particularly BMI cut-offs and the median age threshold, may differ in other racial and ethnic groups. Although an exploratory analysis using WHO BMI categories showed similar trends, this does not replace the need for validation in more diverse populations.

Fourth, as a retrospective study, we were unable to include and control for several crucial post-injury variables that are known to influence the development of CAI, such as the type and duration of bracing, the initiation and adherence to formal physiotherapy, pre-injury activity level, and the timing of return to sport. The nomogram, therefore, provides a baseline risk estimate based on acute injury characteristics but cannot account for the dynamic and modifying effects of subsequent treatment and behavior. Future prospective studies should be designed to capture these variables for a more complete prognostic model.

Fifth, the management of patients was not strictly standardized or randomized, introducing potential confounding by indication. While a general care pathway was in place, individual physician and patient decisions could have influenced outcomes in ways we could not measure.

Sixth, the heavy reliance on MRI findings for the primary nomogram presents a major feasibility challenge in many primary care and emergency department settings. We have attempted to address this with the exploratory development of a simplified clinical model, but this model is less accurate. A formal assessment of the cost-effectiveness and clinical workflow impact of using the MRI-based nomogram vs. a clinical prediction rule is an essential area for future health services research.

Finally, our decision to dichotomize age at its median represents a trade-off between statistical optimality and clinical usability. Our exploratory RCS analysis confirmed that the age-CAI relationship is non-linear (Wald test for non-linearity, *P* = 0.031) and that continuous modeling modestly improves discriminative performance (C-index 0.835 vs. 0.831). We retained the dichotomized model because a binary threshold (≤23 vs. >23 years) is easier to apply in acute care settings. Nonetheless, this simplification inevitably results in some loss of prognostic information. Future studies with larger samples should adopt continuous modeling approaches and consider electronic or web-based implementations to maintain both accuracy and clinical usability.

## 5 Conclusion

This study successfully derived and internally validated a preliminary nomogram for predicting the onset of chronic ankle instability (CAI) following an initial lateral ankle sprain (LAS). The model incorporates five acute-phase prognostic factors: age ≤23 years, BMI ≥24 kg/m^2^, PTFL injury, large talar bone marrow lesion, and Grade 2 tibiotalar joint effusion. Although the model demonstrated promising predictive accuracy in our derivation cohort, its clinical utility remains constrained by the absence of external validation, the potential for MRI-related selection bias, and the homogeneity of the development sample. This tool should be viewed as a foundational step for future research, which must prioritize rigorous external validation and feasibility testing before any consideration of clinical adoption. The identification of these acute MRI-based risk factors also provides new insights into the early pathophysiology of CAI and may inform future mechanistic studies.

## Data Availability

The original contributions presented in the study are included in the article/Supplementary Material, further inquiries can be directed to the corresponding author.
